# Functionalized Nanogels with Endothelin-1 and Bradykinin Receptor Antagonist Peptides Decrease Inflammatory and Cartilage Degradation Markers of Osteoarthritis in a Horse Organoid Model of Cartilage

**DOI:** 10.3390/ijms23168949

**Published:** 2022-08-11

**Authors:** Aurélie Cullier, Frédéric Cassé, Seng Manivong, Romain Contentin, Florence Legendre, Aracéli Garcia Ac, Pierre Sirois, Gaëlle Roullin, Xavier Banquy, Florina Moldovan, Lélia Bertoni, Fabrice Audigié, Philippe Galéra, Magali Demoor

**Affiliations:** 1 Normandie University, UNICAEN, BIOTARGEN, 14000 Caen, France; 2 Research Center of CHU Sainte Justine, Montreal, QC H3T 1C5, Canada; 3 Faculty of Dentistry, Université de Montréal, Montreal, QC H3T 1J4, Canada; 4 Faculty of Pharmacy, Université de Montréal, Montreal, QC H3T 1J4, Canada; 5 TransMedTech Institute (NanoBio Technology Platform), Montreal, QC H3T 1J4, Canada; 6 Department of Microbiology and Immunology, Faculty of Medicine, Université de Laval, Quebec City, QC G1V 4G2, Canada; 7 Center of Imaging and Research on Locomotor Affections in Equines, Veterinary School of Alfort, 14430 Goustranville, France

**Keywords:** chondrocyte, equine model, drug delivery system, bradykinin, endothelin-1, chitosan, hyaluronic acid, interleukin-1 beta, osteoarthritis

## Abstract

Osteoarthritis (OA) is a degenerative and heterogeneous disease that affects all types of joint structures. Current clinical treatments are only symptomatic and do not manage the degenerative process in animals or humans. One of the new orthobiological treatment strategies being developed to treat OA is the use of drug delivery systems (DDS) to release bioactive molecules over a long period of time directly into the joint to limit inflammation, control pain, and reduce cartilage degradation. Two vasoactive peptides, endothelin-1 and bradykinin, play important roles in OA pathogenesis. In this study, we investigated the effects of two functionalized nanogels as DDS. We assessed the effect of chitosan functionalized with a type A endothelin receptor antagonist (BQ-123-CHI) and/or hyaluronic acid functionalized with a type B_1_ bradykinin receptor antagonist (R-954-HA). The biocompatibility of these nanogels, alone or in combination, was first validated on equine articular chondrocytes cultured under different oxic conditions. Further, in an OA equine organoid model via induction with interleukin-1 beta (IL-1β), a combination of BQ-123-CHI and R-954-HA (BR5) triggered the greatest decrease in inflammatory and catabolic markers. In basal and OA conditions, BQ-123-CHI alone or in equimolar combinations with R-954-HA had weak pro-anabolic effects on collagens synthesis. These new nanogels, as part of a composite DDS, show promising attributes for treating OA.

## 1. Introduction

Osteoarthritis (OA) affects over 500 million people worldwide and leads to the loss of health-related quality of life loss [1]. OA is a complex and debilitating disease of the entire joint caused by mechanical, inflammatory, and metabolic factors. OA is characterized by a progressive onset of pain in the joint, swelling, articulation stiffness, and loss of joint mobility. In a healthy joint, hyaline articular cartilage is composed of a main cell type, the chondrocytes, and an abundant extracellular matrix (ECM) mainly made up of type II collagen and aggrecan, which confer joint tissues with viscoelasticity and resistance to compressive forces, providing a smooth lubricated surface for articulation and facilitating the transmission of loads with a low-friction coefficient [2,3]. The pathogenesis of OA relies on inflammation that affects all the components of the joint, notably the articular cartilage, which is entirely degraded at the latest stage of the disease [4]. In the early stage of OA, tissue homeostasis is disrupted, and the ECM composition and organization are altered [5]. Although chondrocytes transiently increased the ECM synthesis and exhibit a higher proliferation, the final outcomes of OA, such as chondrocyte apoptosis and cartilage total destruction, are ineluctable [6]. Indeed, matrix metalloproteinases (MMP) and aggrecanases are overexpressed and progressively induce cartilage degradation. This microenvironment then leads to the activation of inflammation-induced and stress-induced signaling pathways and the secretion of pro-inflammatory cytokines such as IL-1β and TNF-α, also contributing to cartilage degradation [7]. Gradually, the intra-articular space is reduced, and the bone ends come into contact. Many biological and mechanical factors, such as metabolic disorders, aging obesity, or micro- and macro-injuries, contribute to the development of OA, which is characterized by synovial inflammation, focal cartilage loss, osteophyte formation, and subchondral bone sclerosis. Furthermore, due to the absence of vascularization, cartilage is exposed to a hypoxic environment, giving it a poor intrinsic capacity for regeneration.

Animals are not spared from this disease; horses in particular spontaneously develop OA. In sport and racehorses, cartilage injuries are responsible for 60% of lameness, which can abruptly end a racehorse’s sporting career and lead to economic losses [8,9]. As in humans, OA can also occur late in older animals [10]. Furthermore, human and equine articular cartilage share structural and compositional similarities, especially in their cellular and biochemical compositions [8,11]. The horse is therefore a pertinent model for the study of osteoarticular disorders.

There are several OA phenotypes, making the disease difficult to treat. To date, no approved drugs can stop the progression of OA either in humans or in animals. In the early stage of OA, disease-modifying OA drugs (DMOADs) can be used to preserve the joint, but they cannot regenerate the cartilage or halt the evolution of the disease [12]. These first-line treatments are symptomatic background treatments. A physic approach, such as balance training, the use of brace, and the loss of weight, can be considered to ease the symptoms of OA [13]. When symptoms become more severe, non-steroidal anti-inflammatory drugs or other painkillers can be given orally. Hyaluronic acid (HA) or corticosteroids can also be injected intra-articularly. As a last resort, when symptoms are too disabling, joint replacement with a prosthesis may be considered in humans [14]. Current treatments, such as non-steroidal anti-inflammatory drugs or corticosteroids, mainly aim to reduce pain and inflammation [15]. In this context, orthobiological strategies are gaining increasing attention. Orthobiologics are substances (biological molecules) used to treat injuries. For OA, these substances can include HA, corticosteroids, or platelet-rich plasma (PRP) that are injected directly into the injured joint for local treatment [16,17]. Unfortunately, most orthobiological treatments are temporary, and their effect tends to disappear over time [18]. Today, one important challenge in treating OA is to develop new orthobiological strategies that can release therapeutic molecules over a period of time directly into the joint to improve their effectiveness and prolong their retention in the joint while limiting side effects.

Several types of polymers can be used as constituents in drug delivery systems (DDS) including HA, cellulose, or chitosan (CHI). Recent studies have demonstrated the biocompatibility of nanogels combining HA and CHI, which can be used as an intra-articular DDS [19]. HA, a non-sulfated glycosaminoglycan, is widely injected intra-articularly to lubricate cartilage and improve joint movement and tends to recreate a favorable environment for cartilage homeostasis [20]. CHI is a biocompatible and biodegradable synthetic polymer with a structure similar to cartilage glycosaminoglycans. CHI has important chondroprotective effects and also increases HA production [21,22]. However, OA is also characterized by a high inflammatory component involving several types of molecules, including endothelin-1 (ET-1) and bradykinin (BK) [23,24]. ET-1 is a potent vasoconstrictor peptide, exerting its biological activity via two G protein-coupled receptors (GPCR), type A (ET_A_) and B (ET_B_) [25,26]. In articular chondrocytes, the ET_A_ receptor is the predominant receptor type, by which ET-1 triggers nitric oxide (NO) production, upregulates MMP-1,-13 synthesis, and inhibits proteoglycans synthesis [27,28,29]. BK, an endogenous vasodilator peptide known to have potent pro-inflammatory effects, acts on two GPCRs, the B_1_ and B_2_ receptor (BKB_1_ and BKB_2_) [24,30]. BK plays a role in the pathogenesis of OA and contributes to cartilage degradation [31]. The ET_A_ and the BKB_1_ receptors are thus interesting targets for the development of new therapeutics to reduce cartilage degradation, pain, and inflammation in OA. Accordingly, studies have shown that BQ-123 and R-954, two peptide antagonists of ET_A_ and BKB_1_, respectively, decrease cartilage degradation and nociception in a rat model of OA [24].

In this regard, the functionalization of nanogels with a type A endothelin receptor antagonist (BQ-123) or a type B_1_ bradykinin receptor antagonist (R-954) holds promise for simultaneously preventing cartilage destruction, decreasing pain and lubricating the affected joint.

The aim of this study was to elucidate the effects of nanogels composed of CHI or HA functionalized with the peptide antagonists BQ-123 and/or R-954 on equine articular chondrocytes (eACs) cultured as previously described [3,32,33,34]. The biocompatibility of these nanogels was first characterized using toxicity assays, mitochondrial activity, and proliferation analysis on OA chondrocyte model via induction with IL-1β. The effects of the two nanogels BQ-123-CHI and R-954-HA on the metabolism and phenotype of chondrocytes was then evaluated on a cartilage organoid model based on a study of hyaline, catabolic, and inflammatory biomarkers. It can be supposed that nanogels composed of BQ-123-CHI and R-954-HA could protect cartilage from inflammation and the degradation processes that occur during OA.

## 2. Results

### 2.1. Nanogels Have No Cytotoxic Effects, Do Not Alter Viability, and Sustain Metabolic Activity and Proliferation of Equine Articular Chondrocytes

**Figure 1:**
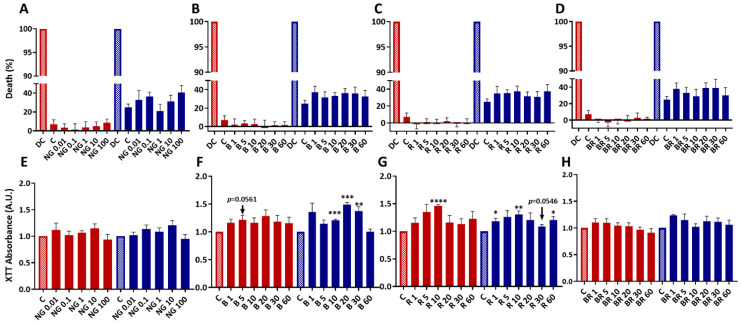
Nanogels have no cytotoxic effects and do not alter the viability and proliferation of equine articular chondrocytes (eACs). eACs were amplified and seeded at the third passage in monolayer (20,000 cells/cm^2^). At 80% confluence, cells were treated with nanogels, namely NG at 0.01, 0.1, 1, 10, or 100 μg/mL; BQ-123-CHI (B); R-954-HA (R); and BR at 1, 5, 10, 20, 30, or 60 nM, and then incubated in normoxia **(red)** or in hypoxia **(blue)** for 72 h. Control (without nanogels) and death control (Triton X100-induced death) were included. At the end of the incubation period, the levels of adenylate kinase (cytotoxicity) (**A**–**D**) and formazan (XTT) (**E**–**H**) were measured in the media. Data are represented as histograms (*n* = 3) and Student’s *t*-tests were used to determine treatments that differ significantly from the control (cytotoxicity/XTT) (* *p* < 0.05, ** *p* < 0.01, *** *p* < 0.001, **** *p* < 0.0001). Results were normalized to the death control (for cytotoxicity) or control (from XTT). NG, Non-functionalized nanogel; B, BQ-123-CHI; R, R-954-HA; BR, equimolar combination of BQ-123-CHI and R-954-HA; C, control; DC, death control; A.U., arbitrary units.

We have previously demonstrated that eACs grown in monolayers, like human chondrocytes, lose their specific expression profile during dedifferentiation achieved by passages and acquire an elongated fibroblast-like morphology [35,36]. Chondrocytes dedifferentiate rapidly; their gene expression is modified from the first passage in monolayer culture [37,38]. The dedifferentiation-associated events are similar to the chondrocyte phenotypic shift that occurs during OA [39]. Thus, dedifferentiated chondrocytes are accepted as a relevant in vitro model to assess the therapeutic potential of innovative strategies that aim to improve OA treatments. Herein, we used dedifferentiated eACs to assess the biocompatibility and efficacy of the nanogels. eACs were seeded and treated at 80% confluence with four different nanogel formulations at concentrations ranging from 0.01 to 100 µg/mL for non-functionalized CHI-HA nanogels (NG) and from 1 to 60 nM of peptide for BQ-123-CHI (B), R-954-HA (R), and the BR combination (Figure 1). Functionalized nanogels size was between 429 and 733 nm with a Polydispersity Index (PdI) of ~0.3–0.6 and zeta potential (ZP) between +47 and +51 mV (Table 1). Peptide release of R-954-HA conjugate in human synovial fluid (hSF) showed 23% release of R-954 after 77 h and even longer for BQ-123-CHI; thus, the calculated peptide release from the nanogel complexes was estimated to be at least of 39 days for both peptides [40].

Cytotoxicity was evaluated 72 h after treatment in normoxia and in hypoxia (the physioxic environment of chondrocytes). The percentage of death was higher in hypoxic conditions than in normoxic conditions. However, regardless of the treatment, there were no significant differences with the control condition in either normoxia or hypoxia. Whatever the nanogel formulation (NG, B, R, BR), the concentration, and the oxic condition, nanogels were not cytotoxic for eACs (Figure 1A–D).

We then evaluated the metabolic activity after 24, 48, and 72 h of nanogel treatment, with the same formulations and concentrations used previously. After 72 h of treatment, there were no significant differences between NG concentrations and the control regardless of the oxic conditions (Figure 1E). Similar results were observed at 24 h and at 48 h except for the 10 and 100 μg/mL NG formulations (in normoxia and hypoxia, respectively), which showed a statistically significant increase (Supplementary Materials Figure S1).

In hypoxia, 72 h after treatment with 10, 20, and 30 nM B nanogels, eACs metabolic activity increased significantly compared with the control (1.2-, 1.5-, and 1.4-fold higher, respectively) (Figure 1F). This increase was also observed after 48 h, whereas only 20 and 30 nM B nanogels led to an increase of eACs metabolic activity after 24 h (Supplementary Materials Figure S1). Furthermore, the highest concentration, 60 nM B, did not modulate the metabolic activity compared with the control (Figure 1F and Supplementary Materials Figure S1). In normoxia, a similar pattern was obtained with the different culture times but with a significant increase only after 24 h of treatment. Under all conditions, including normoxia and hypoxia, the 20 and 30 nM concentrations showed the maximum effect of B on eACs metabolic activity. The 10 nM R nanogel significantly increased eACs metabolic activity after 24 and 48 h of treatment (1.5- and 1.7-fold increase, respectively) in hypoxia. The same trend was observed after 72 h of treatment. Again, in hypoxia, 1, 10, and 60 nM R nanogels induced a significant increase in the metabolic activity 72 h after treatment. At the same time, in normoxia, 5 and 10 nM R nanogels enhanced eACs viability by a factor of 1.2 and 1.3 times compared with the control (Figure 1G). Under all conditions, including normoxia and hypoxia, the 5 and 10 nM R concentrations gave the maximum effect of R nanogels on eACs metabolic activity.

Regarding BR nanogels, the BR combination did not induce synergistic effects. At the end of incubation period (72 h), no significant differences were found between the different BR concentrations in normoxia or hypoxia (Figure 1H). However, 1 and 5 nM BR seemed to increase metabolic activity compared with the control regardless of the oxic conditions.

All these results showed a maximum effect on cellular activity for 20 and 30 nM BQ-123-CHI and 5 and 10 nM R-954-AH.

The 5 nM and 30 nM R-954-HA and BQ-123-CHI nanogel formulations were selected for the subsequent studies using functionalized nanogels either alone or in combination (BR). CHI-HA nanogels at 0.1 and 10 μg/mL were selected, corresponding also to the concentration of CHI or HA in the 5 and 30 nM B or R formulations, respectively.

**Figure 2:**
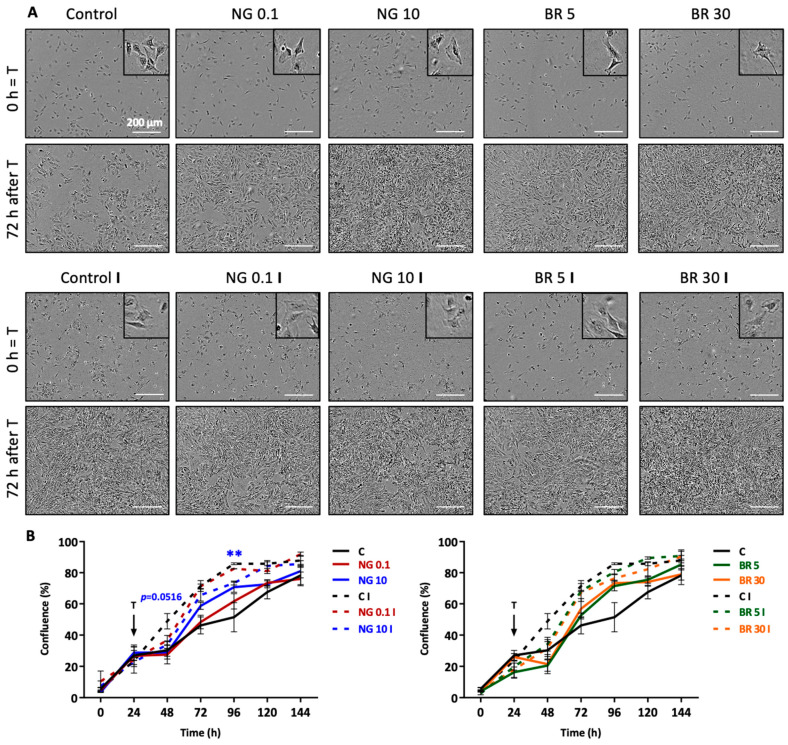
Effects of nanogels on the proliferation of equine articular chondrocytes (eACs). eACs were amplified and seeded at the third passage in monolayer (20,000 cells/cm^2^). Then, 24 h after seeding, cells were treated with nanogel formulations in the presence of 5% FBS: 0.1 and 10 μg/mL NG, 5 and 30 nM BR, with or without IL-1β (10 ng/mL). Proliferation was monitored using IncuCyte^®^. At the end of the incubation period, confluence was analyzed with ImageJ software (ImageJ 1.35c, Wayne Rasband, National Institutes of Health, Bethesda, MD, USA). Pictures were taken the day of treatment and every 24 h (scale bar 200 μm) (**A**). Data are represented as curves (*n* = 3) (**B**). Student’s *t*-tests (** *p* < 0.01) were used to compare each treatment with the control (**C**) and each treatment including IL-1β with the control IL-1β (C I). NG, non-functionalized nanogel; BR, equimolar combination of BQ-123-CHI and R-954-HA; C, control; I, IL-1β.

To complete the biocompatibility study of the nanogels, eACs proliferation and cell morphology were monitored in normoxia using live imaging with IncuCyte^®^ technology (IncuCyte S3 microscope and IncuCyte 2021A software, Sartorius, Göttingen, Germany). Cells were seeded and treated after 24 h with the two chosen concentrations of formulation mentioned above (0.1 and 10 μg/mL NG, 5 and 30 nM BR), with or without IL-1β (10 ng/mL). The cells were cultured in the presence of IL-1β, the condition used in subsequent studies, to mimic the pro-inflammatory environment occurring during OA. Firstly, eACs were adherent cells and had a typical polygonal shape (Figure 2A). In the presence of IL-1β, maximum confluence was reached more rapidly than in the absence of IL-1β (Figure 2B). Indeed, 48 h after treatment (i.e., 72 h after the beginning of the culture), 65% confluence was observed when eACs were cultured in the presence of IL-1β compared with 40% for the control without IL-1β. The 10 μg/mL NG (45% confluence) and 5 and 30 nM BR nanogel treatments (70% of confluence) (Figure 2B) seemed to increase the eACs proliferation compared with the control in the absence of IL-1β. Moreover, in the presence of IL-1β, all treatments did not increase eACs proliferation. 

**Figure 3:**
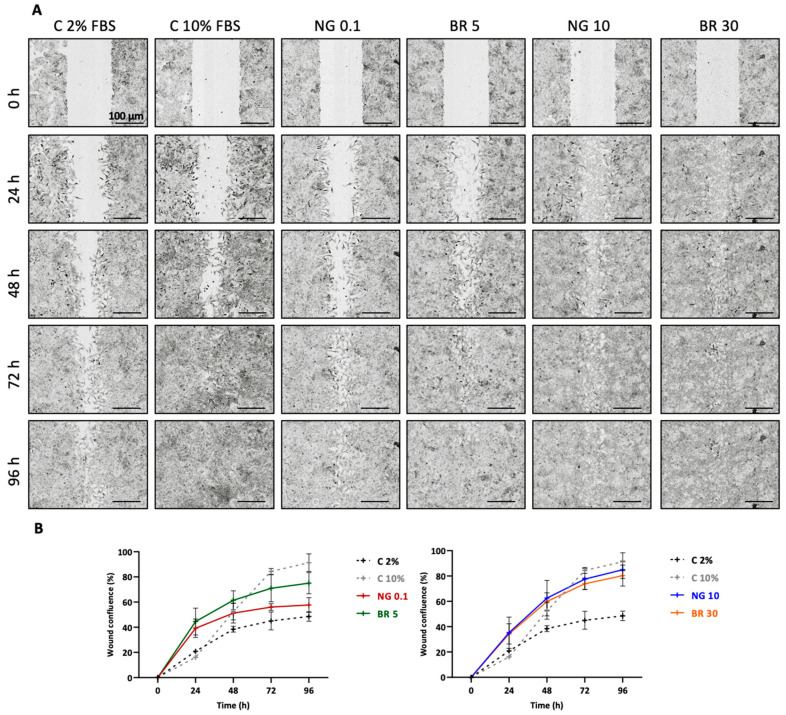
Effects of nanogels on wound filling. Equine articular chondrocytes (eACs) were amplified and seeded at the third passage in monolayer (20,000 cells/cm^2^). At 100% confluence, wound filling was assayed in each well, and nanogel treatments were added to cells NG at 0.1 and 10 μg/mL and BR at 5 and 30 nM (in the presence of 2% FBS (C2%)), and proliferation was followed by IncuCyte^®^. The cells were also incubated in the presence of 10% FBS (C10%). At the end of the incubation period, confluence and wound area were analyzed using ImageJ software. Pictures were taken on the day of treatment and every 24 h (scale bar 100 μm) (**A**). Data are represented as curves (*n* = 3) (**B**). NG, non-functionalized nanogel; BR, equimolar combination of BQ-123-CHI and R-954-HA; C, control.

In parallel, the effects of nanogels on the proliferation/migration of eACs were assessed using a wound healing assay and the IncuCyte^®^ technology. In the absence of IL-1β and in normoxia, the wound filled more rapidly when chondrocytes were cultured in the presence of 10% FBS rather than 2% FBS (Figure 3A,B). When chondrocytes were treated with 10 μg/mL NG or 30 nM BR in presence of 2% FBS, they colonized the wound areas in a similar pattern to eACs cultured with the control medium containing 10% FCS. When compared with its non-functionalized counterpart (0.1 μg/mL NG), 5 nM BR seemed to also favor proliferation (Figure 3A,B). Similarly, with IL-1β, non-functionalized nanogels and 5 nM BR appeared to better promote chondrocyte proliferation. In contrast, the 30 BR nanogel treatment did not induce an effect on proliferation/migration compared with the control (statistically insignificant results) (Supplementary Materials Figure S2).

Overall, the 5 nM BR treatment (BQ-123-CHI and R-954-HA at 5 nM) seemed to sustain metabolic activity and to favor proliferation of eACs when cultured in the basal condition (i.e., without IL-1β) and to a lesser extent in the presence of IL-1β. Thus, 5 nM BR-functionalized nanogels appeared to promote chondrocyte proliferation compared with the non-functionalized CHI-HA nanogels.

### 2.2. Characterization of the Organoid Model of Osteoarthritic Cartilage Tissue

**Figure 4:**
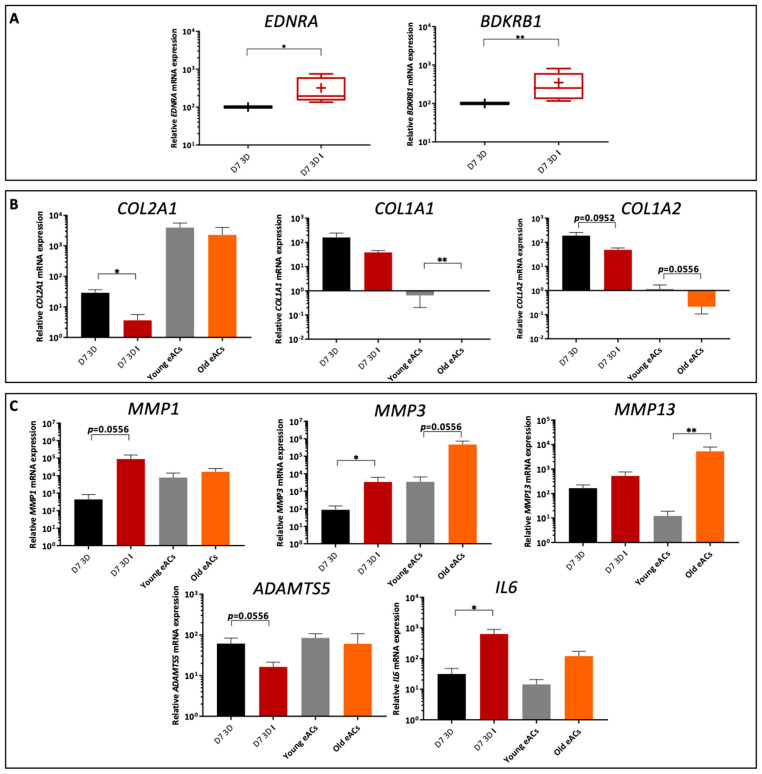
Gene expression analysis in an equine OA organoid model. Equine articular chondrocytes (eACs) at the third passage were seeded in type I/III collagen sponges and then incubated for 7 days in hypoxia in the absence or presence of IL-1β (I) (10 ng/mL). Transcript expression of endothelin and bradykinin receptor (**A**), some matrix (**B**), and catabolic and inflammatory (**C**) markers are shown in arbitrary units. Mann–Whitney tests were used to test for differences between treatments with or without IL-1β or between young and old eACs; *n* = 5 (* *p* < 0.05, ** *p* < 0.01) D7 3D, control; D7 3D I, control IL-1β; Young eACs, young eACs grown in monolayer P0 (2–5 years); Old eACs, old eACs grown in monolayer P0 (24–26 years).

The involvement of ET-1 and BK in the pathogenesis of OA has been demonstrated in humans and in rats, for which an inflammatory environment and pro-inflammatory cytokines such as IL-1β increased the expression of their receptors in chondrocytes [28,31]. In the equine model, when eACs were cultured in organoids, IL-1β (10 ng/mL) induced a significant 3.5-fold increase in the ET_A_ (*EDNRA*) and BKB_1_ (*BDKRB1*) receptor mRNA expression compared with the control (Figure 4A).

To evaluate the efficacy of the nanogel formulations, an OA cartilage organoid model was used. For this purpose, chondrocytes dedifferentiated after three passages were seeded in collagen sponges and cultured in 3D and hypoxia for 7 days, and IL-1β was added to mimic an inflammatory environment. To characterize this organoid model, the mRNA expression of various markers was then analyzed and compared with chondrocytes cultured in monolayers (passage 0) from young (2–5 years) and old horses (24–26 years). Firstly, for the matrix markers of hyaline cartilage, results show a significant decrease in *COL2A1* mRNA levels (7.5 times) in the inflammatory model compared to the control condition (Figure 4B). Secondly, for type I collagen, a fibrotic cartilage marker, both *COL1A1* and *COL1A2* mRNA levels were higher in the 3D OA model than in young and old eACs primocultures. Moreover, expression levels were five times lower in inflammatory conditions than in control conditions as well as for the old eACs primocultures (Figure 4B).

Regarding the catabolic markers, the steady-state amounts of metalloproteinases *MMP1*, *MMP3*, and *MMP13* were higher by a factor of 250, 30, and 3.33, respectively, in the inflammatory environment (D7 3D I condition) compared with the control. The *MMP3* and *MMP13* mRNA levels were increased in old eACs compared with the young eACs (respectively 166 and 500 times more expressed in the old eACs). Conversely, *ADAMTS5* decreased by 4-fold in the inflammatory environment (D7 3D I condition) compared with the control condition, and expression levels did not seem to differ between the young and old eACs (Figure 4C). Finally, the inflammatory marker *IL6* was 20 times higher in the induced inflammatory environment than in the control, with similar trends for young and old eACs.

**Figure 5:**
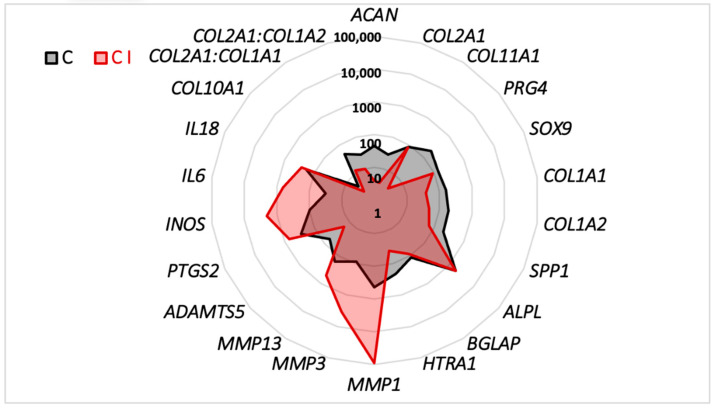
Comparison of mRNA expression in organoid cultures of equine articular chondrocytes (eACs) in the presence or absence of IL-1β. eACs at the third passage were seeded in type I/III collagen sponges and then incubated for 7 days in hypoxia in the presence or absence of IL-1β (I) (10 ng/mL). The radar chart indicates the normalized mRNA expression levels of some matrix, catabolic, and inflammatory markers. The mRNA levels were estimated using RT-qPCR after normalization with respect to the *ACTB* and *TUBA* reference genes. C, control; C I, control IL-1β.

**Figure 6:**
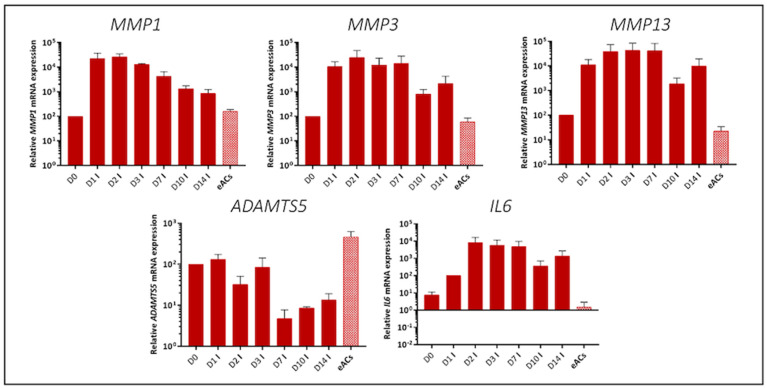
Gene expression analysis of equine articular chondrocytes (eACs) cultured with IL-1β according to 14-day kinetic assay. eACs at the third passage were seeded in type I/III collagen sponges and then incubated in presence of IL-1β (I) (10 ng/mL) for 0, 1, 2, 3, 7, 10, and 14 days. Transcripts of some catabolic and inflammatory markers are shown in arbitrary units. Mann–Whitney tests were used to determine treatments that differ significantly from initial level (D0); *n* = 3. eACs, equine articular chondrocytes (passage P0); I, IL-1β.

In the inflammatory environment (D7 3D I condition), eACs show low expression of type II collagen, which is specific to hyaline cartilage, and high expression of type I collagen (atypical isotype). The addition of IL-1β reduced the expression of these matrix molecules and increased the expression of catabolic and inflammatory markers characteristic of OA (Figure 5). According to a 14-day kinetic assay, IL-1β increased the mRNA levels of *MMP1*, *MMP3*, *MMP13*, and *IL6* from the first day and maintained the levels for up 7 days. After 10 days of culturing, mRNA expression levels dropped (Figure 6).

### 2.3. Functionalized Nanogels Decrease the Inflammatory and Catabolic Cartilage Markers

We therefore used this 7-day organoid model to evaluate the effectiveness of functionalized nanogels. Organoids obtained with five strains of eACs were incubated with NG at 0.1 and 10 μg/mL; B, R, and BR at 5; and 30 nM IL-1β and cultured in hypoxia.

**Figure 7:**
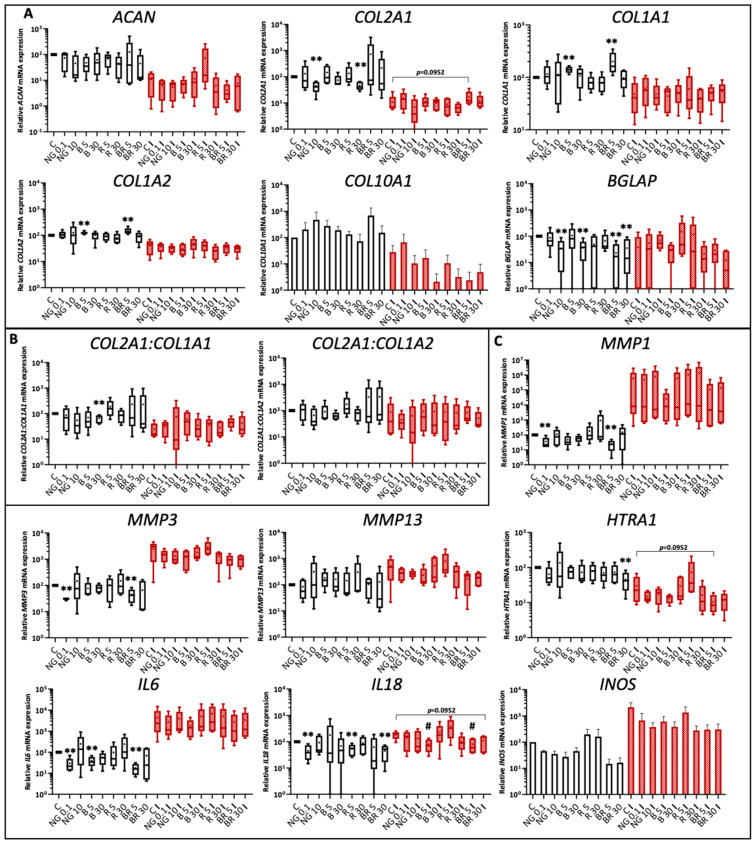
Gene expression analysis of equine articular chondrocytes (eACs) cultured with nanogels and IL-1β. eACs were seeded in type I/III collagen sponges and then incubated for 7 days in hypoxia in the absence (C) or presence of nanogel formulations (NG at 0.1 and 10 μg/mL and B, R, and BR at 5 and 30 nM) and in the absence **(black)** or presence **(red)** of IL-1β (C I) (10 ng/mL). Box plots show transcript expression of some matrix (**A**), catabolic, and inflammatory (**C**) markers in arbitrary units (*n* = 5 except for *COL10A1* and *INOS*, *n* = 3). The *COL2A1:COL1A1* and *COL2A1:COL1A2* ratios are also given (**B**). Mann–Whitney tests were used to determine treatments that differ significantly from C (*) and C I (#) (# *p* < 0.05, **, *p* < 0.01). NG, non-functionalized nanogel; B, BQ-123-CHI; R, R-954-HA; BR, equimolar combination of BQ-123-CHI and R-954-HA; C, control; I, IL-1β.

For the matrix markers, *ACAN* mRNA levels tended to decrease in all conditions compared with the control in absence of IL-1β except for 5 nM BR, which increased slightly (Figure 7A). In the inflammatory condition, *ACAN* expression increased 8-fold with 5 nM R compared with the control. In basal conditions, *COL2A1* steady-state amounts were 2-fold higher with 0.1 μg/mL NG, 5 nM B, and 5 nM R; 3-fold with 30 nM BR; and 7-fold with 5 nM BR compared with the control. However, the 10 μg/mL NG and 30 nM R nanogel treatments showed 2-fold decrease *COL2A1* mRNA levels relative to the control. In the presence of IL-1β, the 5 nM BR nanogel showed slightly (but not significantly) higher *COL2A1* mRNA levels than the control (*p* = 0.0952). Regarding the expression of type I collagen, *COL1A1* increased significantly by 1.4-fold and 2-fold with 5 nM B and 5 nM BR nanogel treatments, respectively, relative to the control without IL-1β. The 5 nM B and 5 nM BR nanogel treatments, respectively, showed a significant 1.2 and 1.4-fold increase in *COL1A2*. Under inflammatory conditions, non-significant changes were observed. Without the inflammatory component, the ratios *COL2A1:COL1A1* and *COL2A1:COL1A2* doubled or even tripled with the 5 nM BR and 30 nM BR compared with the control and the non-functionalized nanogels (0.1 and 10 μg/mL NG). In the presence of IL-1β, little decrease or no effect was observed (Figure 7B). Type X collagen, *COL10A1*, a hypertrophic marker, increased 7-fold with the 5 nM BR but not in the 30 nM BR nanogels. In the presence of IL-1β, the *COL10A1* mRNA level decreased strongly in 5 nM and 30 nM BR nanogel treatments (expression 15 times lower than in the control) (Figure 7A). Other cartilage-associated molecule markers were also analyzed (Supplementary Materials Figure S3). For example, *COL11A1* steady-state amounts were significantly higher in the non-functionalized (0.1 μg/mL), 5 nM B, and 5 nM BR treatments (2.2, 2, and 2.5 times higher, respectively) (Supplementary Materials Figure S3). With IL-1β, the *COL11A1* mRNA level decreased 2.5-fold in 0.1 μg/mL NG but not in 5 nM BR. Finally, *BGLAP*, a marker of bone phenotype, was evaluated. In particular, *BGLAP* showed lower expression in the 10 μg/mL NG, 30 nM B, and 5 and 30 nM BR nanogel treatments, with the lowest expression in the 5 nM BR nanogel (five times significantly lower than the control and the non-functionalized 0.1 μg/mL NG nanogel). The same trend was observed under inflammatory conditions (Figure 7A). 

Regarding markers involved in inflammation and degradation, the non-functionalized (0.1 μg/mL) and 5 nM BR combination nanogel treatments showed significantly decreased expression of *MMP1* and *MMP3* in the basal condition compared with the untreated condition (2.5 and 3.3 times less for *MMP1* and 3.3 and 2 times less for *MMP3*, respectively). Under inflammatory conditions, the 5 and 30 nM BR nanogels led to lower steady-state amounts of the three MMPs studied compared with the IL-1β control alone (5-fold less for *MMP1*, 3-fold less for *MMP3*, and 3.3-fold less for *MMP13* in 5 nM BR). Inflammatory markers such as *IL6* and *IL18* also showed modified expression with the nanogels. When cultures were conducted without IL-1β, the 0.1 μg/mL NG, 5 nM B, and 5 nM BR nanogels gave significantly reduced *IL6* mRNA levels. Likewise, 0.1 μg/mL NG, 5 nM R, and 30 nM BR nanogel treatments showed significantly reduced *IL18* transcript levels. The greatest decrease in *IL6* mRNA levels (5-fold) was observed in 5 nM BR, whereas that of *IL18* was reduced by half in the presence of 30 nM BR. Under inflammatory conditions, slight effects were observed for *IL6*, but *IL18* transcript levels were significantly halved in 5 nM B and 5 nM BR nanogels. *HTRA1* expression was also three times lower in the 30 nM BR nanogel in the basal condition and halved in 5 nM and 30 nM BR nanogels in inflammatory condition. Transcript levels of *INOS* (NO synthase inducible gene), another important marker of inflammation, also decreased by almost 7-fold in the 5 and 30 nM BR combination nanogels compared with the control (to a lesser extent when compared with the non-functionalized nanogels), with or without IL-1β (Figure 7C). Other catabolic markers such as *ADAMTS5* were almost two times lower at the transcript level in the 30 nM BR nanogel under basal and inflammatory conditions. There was little variation in *IL1B* mRNA levels (Supplementary Materials Figure S3). The combination BR nanogel treatment, particularly at 30 nM, significantly reduced the mRNA level of *P65* in the basal condition by a factor of 1.5. The 5 nM BR nanogel treatment also showed decreased *P65* expression by 1.2-fold (not statistically significant). No effect was observed on *P53* mRNA levels. *KI67*, a marker of proliferation, showed reduced expression with the non-functionalized nanogels and with the 5 nM B, 30 nM R, and 5 and 30 nM BR nanogels under inflammatory conditions (Supplementary Materials Figure S3).

**Figure 8:**
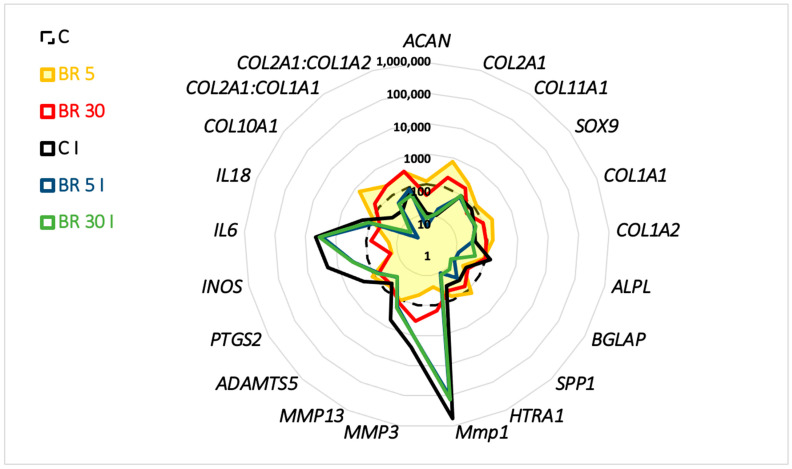
Comparison of mRNA expression in organoid cultures of equine articular chondrocytes (eACs) in the presence or absence of IL-1β and the combination nanogel BR. eACs were seeded in type I/III collagen sponges and then incubated for 7 days in hypoxia in the presence of IL-1β (I) (10 ng/mL) and BR at 5 and 30 nM. The mRNA levels were estimated using RT-qPCR after normalization with respect to the *ACTB* and *TUBA* reference genes. The transcript expression levels of some matrix, catabolic, and inflammatory markers are shown in a radar chart. BR, equimolar combination of BQ-123-CHI and R-954-HA; C, control; I, IL-1β.

The 5 nM BR nanogel induced a slight increase in the mRNA levels of matrix markers. Furthermore, BR nanogel limited the steady-state amount of markers of inflammation and degradation and did not induce a hypertrophic or osteoblastic phenotype (Figure 8). In addition, the functionalized BR nanogels showed a higher efficiency than the non-functionalized CHI-HA nanogels.

**Figure 9:**
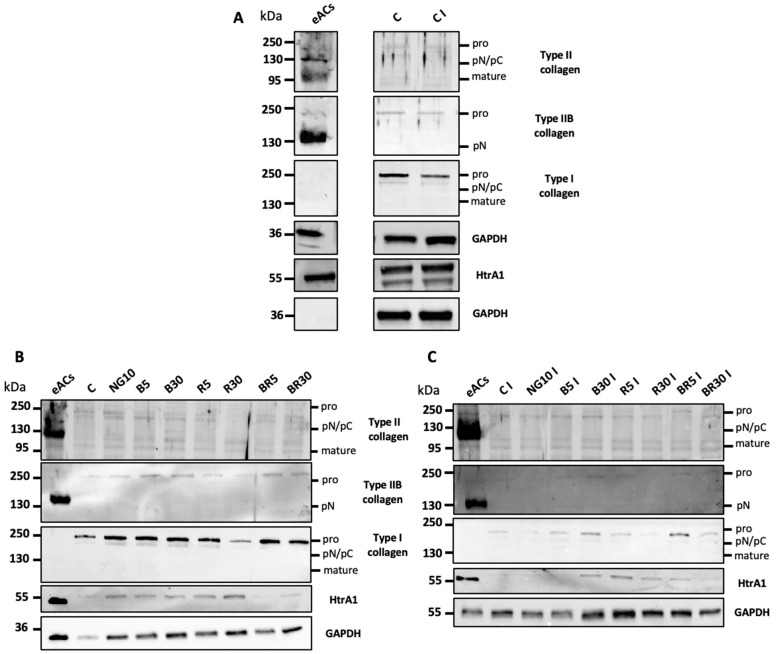
Effects of nanogels on protein expression of type I and II collagen and HtrA1 in organoid cultures of equine articular chondrocytes (eACs). eACs at the third passage were seeded in type I/III collagen sponges and were incubated for 7 days in hypoxia in the absence (**C**) or presence of nanogels formulations (NG at 0.1 and 10 μg/mL and B, R, and BR at 5 and 30 nM) and in the absence (**B**) of presence of IL-1β (C I) (**C**) (10 ng/mL). The comparison between basal conditions and IL-1β is also shown (**A**). The molecular weight (kDa) is shown on the left side of the panels. Representative blots from different eACs strains are shown (*n* = 3). NG, non-functionalized nanogel; B, BQ-123-CHI; R, R-954-HA; BR, equimolar combination of BQ-123-CHI and R-954-HA; C, control; I, IL-1β; kDa, kilodaltons.

The expression of proteins reflecting the chondrocyte phenotype was also assessed in organoid cultures: type II an IIB collagen isoforms, expressed by mature chondrocytes, and type I collagen and HtrA1, which are expressed by fibrotic chondrocytes with an OA profile. Chondrocytes cultured in 3D in the presence or absence of IL-1β showed low expression levels of type II and IIB collagens. Type I collagen was more weakly expressed in inflammatory conditions. The expression of the protease HtrA1 did not seem to vary between the two conditions (Figure 9A and Supplementary Materials Figures S4 and S6).

After 7 days of culture and under basal conditions, chondrocytes seeded after the third passage in 3D scaffolds synthesized a low level of type II collagen corresponding to immature forms (pro, pN, and pC). Similarly, the synthesis of type IIB collagen was revealed by a weak signal at 250 kDa. In contrast, the pro-form of type I collagen at 250 kDa was also expressed in this 3D chondrocytes cultures.

Considering the three isoforms (pro, pN/pC, and mature) of collagens, 5 and 30 nM B increased both types of collagens II and I compared to NG, and the same trend is observed when B is added simultaneously with R at equimolar combinations. In the presence of IL-1β, the effects seemed preserved with these same formulations. Regardless of the concentration, B and its combination with R weakly increases the synthesis of matrix components without any specific effects.

With regard to the serine protease HtrA1, the BR formulation at 5 and 30 nM seemed to reduce its expression under basal conditions and compared with the control and other formulations (Figure 9B,C and Supplementary Materials Figures S5 and S6).

### 2.4. Non-Functionalized and Functionalized Nanogels Triggered a Decrease of Nitric Oxide Synthesis

**Figure 10:**
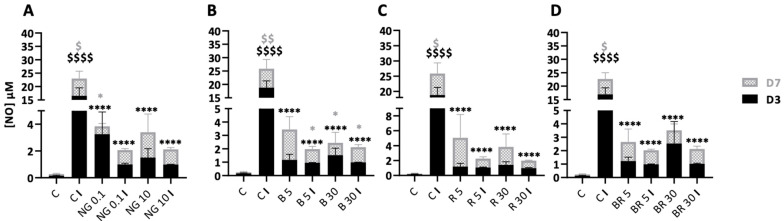
Nanogels decrease nitric oxide (NO) concentrations induced by IL-1β in organoid cultures of equine articular chondrocytes (eACs). eACs at the third passage were grown in type I/III collagen sponges and then cultured for 7 days in hypoxia in the absence (C) or presence of nanogel formulations (NG at 0.1 and 10 μg/mL (**A**) and B, R, and BR at 5 and 30 nM (**B**, **C**, and **D**, respectively)) and in the absence or presence of IL-1β (C I) (10 ng/mL). The NO concentration was assessed using the Griess method in the organoid culture media after 3 (D3) and 7 days (D7) of incubation. The results are shown as box plots (*n* = 4–6) and statistical analyses using a two-way ANOVA followed by post hoc Tukey tests, which were employed to determine treatments that differ significantly from C ($) or C I (*) (*, $ *p* < 0.05; $$ *p* < 0.01; ****, $$$$ *p* < 0.0001).

Chondrocytes were seeded in 3D and then treated with the different nanogel formulations in the presence or absence of IL-1β for 7 days. NO was measured in the culture media collected after 3 and 7 days of culture. Firstly, IL-1β significantly increased NO synthesis in cartilage tissue organoids at D3 and D7 relative to the control with, respectively, an increase of 124-fold and 58-fold. When the organoids were incubated in the presence of the non-functionalized nanogels in the absence of IL-1β, there was an increase in NO concentration in the presence of 0.1 μg/mL (21-fold at D3, 4.8-fold at D7) and 10 μg/mL (10- and 9-fold at D3 and D7, respectively) relative to the control. However, NO synthesis was much lower in the control than that induced by IL-1β (110 times lower). Interestingly, on D3, in the presence of IL-1β and 0.1 or 10 μg/mL NG, NO synthesis was significantly lower (by a factor of 16.7) than in the IL-1β control condition. At D7, the amount of NO was also decreased with NG with the control IL-1β but was not significant (Figure 10A). Finally, non-functionalized nanogels decreased NO synthesis under inflammatory conditions, i.e., in the presence of IL-1β, to a greater extent on D3 than on D7. Nevertheless, in the absence of IL-1β, the 5 nM B nanogel induced a 7.8- and 24-fold increase in NO concentration, respectively, on D3 and D7 compared with the control. In contrast, with IL-1β, this formulation significantly decreased NO synthesis on D3 and D7 (19.8- and 6.9-fold, respectively) compared with C I. Similarly, NO synthesis was inhibited in the presence of 30 nM B (Figure 10B). R nanogels showed the same trend as B nanogels, with 5 and 30 nM R in the absence of IL-1β being associated with a non-significant increase in NO concentration compared with the control. On the contrary, in inflammatory conditions, NO synthesis decreased by about 17.7 and 19.2 times, respectively, with the R nanogels formulations, after 3 days of culture in hypoxia (Figure 10C). On D3, the combination BR nanogel showed decreased NO concentrations in the presence of IL-1β by approximately 17-fold compared with the IL-1β control condition (C I) (Figure 10D). Overall, nanogel formulations inhibited IL-1β-induced NO synthesis, whatever their concentration and their functionalization.

## 3. Discussion

Here, we developed a new biomaterial composed of two peptides (BQ-123 and R-954 conjugated to the CHI and HA and incorporated into the NG), validated the biocompatibility of these nanogels, and investigated the metabolic, anabolic and catabolic effects on equine articular chondrocytes cultured under different physioxic conditions. This new biomaterial could be used as a new injectable orthobiological strategies to potentially control cartilage regeneration and inflammation in order to manage OA effectively. Current orthobiological treatments are based on emerging therapies such as viral vector expression, gene therapy, PRP injection, and DDS of bioactive molecules or RNA to target multiple actors involved in the OA pathogenesis [41,42,43]. Our new biomaterial could be used to effectively deliver therapeutic molecules directly into the joint in an effective and sustainable manner to reduce inflammation, prevent cartilage degradation, improve nociceptive tolerance, or even promote cartilage regeneration. This approach is based on the previously observed effects, such as the synergistic inhibition of endothelin-1 and bradykinin receptors by two peptides, BQ-123 and R-954, respectively, that can decrease pain and prevent the destruction of cartilage in a surgically induced OA model [24]. CHI- and HA-based nanogels are of interest to improve OA treatments, notably because they could be used as DDS [44,45,46,47]. In this study, CHI- and HA-based nanogels were developed as a drug delivery platform for the concomitant intra-articular administration of BQ-123 and R-954 peptides. Our goal was to test on eACs the biocompatibility and the efficacy of new DDS nanogels composed of CHI and HA conjugated with BQ-123 and R-954 peptides, respectively.

We first assessed the biocompatibility of the different formulations by analyzing cytotoxicity and cell viability/proliferation. None of the formulations, regardless of the dose, had a cytotoxic effect on eACs. Interestingly, we found that combination nanogels with BQ-123-CHI and R-954-HA at 5 nM seem to promote eACs metabolic activity and proliferation, which make them useful for improving chondrocyte metabolism. During OA pathogenesis, mitochondrial dysfunction may have an impact on chondrocyte anabolism and exacerbate oxidative stress and chondrocyte apoptosis, which all contribute to cartilage degeneration [48]. Interestingly, some nanogel formulations positively modulated metabolic activity. Similarly, we found that, with BR combination nanogels, chondrocytes tented to have an increased proliferation. Increased proliferation and metabolic activity of chondrocytes may be a first step toward tissue repair.

This study was performed using cartilage tissue organoids and chondrocytes from horses. The equine model used in this study has a double advantage. It is the animal model with the most significant number of similarities with humans, and the horse develops the disease spontaneously, either early or later in life, like humans [8,11,49]. Additionally, any progress in equine OA treatment may be transferable to humans. Equine cartilage tissue organoid models were incubated in the presence of IL-1β, a major pro-inflammatory cytokine involved in the development of OA [50]. IL-1β can significantly induce an inflammatory and catabolic environment within the organoid model, characterized by an increase in *MMP* expression and a decrease in the expression of matrix markers, such as collagens and aggrecan at mRNA level. IL-1β has no effect on the expression of *ADAMTS5*, an aggrecanase widely involved in cartilage degradation during early OA events. However, its expression is constitutive in both normal and OA cartilage, which is consistent with the results obtained in our model [51,52].

OA is a progressive disease characterized by different sequential events. Following damage lesions, intra-articular inflammation occurs. Then, as a first response, the proliferation of chondrocytes increases, and the composition of the ECM changes and contains mostly an atypical collagen molecule, the type I collagen. Finally, chondrocytes become hypertrophic and senescent and undergo apoptosis, leading to cartilage that is inevitably degraded. In our model, chondrocytes show very low mRNA expression of the transcripts of the matrix components of hyaline cartilage (type II collagen, aggrecan, type XI collagen) in favor of enhanced expression of OA markers, such as serine proteases (*HTRA1*) and metalloproteinases (*MMP1*, *MMP3,* and *MMP13*), suggesting reduced synthesis of a nonetheless fibrous matrix and an increased catabolic activity. The BR combination nanogel appeared to attenuate the deleterious effects of IL-1β by reducing the expression of catabolic and inflammatory markers. These effects were also found at the protein level with, in particular, a low expression of HtrA1, a protease largely involved in the degradation of the ECM, because it induces the expression of MMP-1 and -3, which degrade collagens. HtrA1 is an interesting target for nanogels because high levels of HtrA1 are found in the synovial fluid of OA patients [53]. Indeed, studies have shown that HA can decrease the expression of MMP by interacting with the synoviocytes of the synovial membrane, whereas ET-1 induces an increase in the synthesis of MMP-1 and -13 in chondrocytes. Thus, the modulation of catabolic and inflammatory markers by nanogels combining matrix polymers such as HA and an ET-1 antagonist is a promising approach and may lead to delay the progression of OA.

NO is another important inflammatory mediator involved in OA, and studies show that an increase in NO promotes cartilage degradation [54]. Here, NO strongly increased in media under inflammatory conditions but significantly decreased in the presence of nanogels, without any cumulative effect of the different formulations. However, in non-inflammatory basal conditions, NO expression tended to increase in the presence of the different nanogels. It has been reported that ET-1 modulates the production of NO, and thus, the presence of an ET-1 antagonist, BQ-123, may explain this effect on NO levels [55].

Regarding matrix markers, there was a weak pro-anabolic effect with B, an effect maintained with its equimolar combinations with R. These effects are associated with relatively weak Western blot signals for type II collagen compared to type I collagen. However, it is difficult to compare quantitatively the expressions obtained for both types of collagens because, as we have shown in our previous studies [34], the antibody used against type I collagen presents a much higher titer than the one targeting type II collagen. The weak signals can also probably be explained by the nanogel incubation time, which may be too short. We chose 7 days of culture in 3D because this duration corresponds to the maximum time of action of IL-1β with a high expression of MMPs in particular. After 7 days, the expression of inflammatory and degradation markers drops even in the presence of IL-1β, probably due to internalization of IL-1β receptors. In addition, the eACs in this study were used at passage 3. Therefore, they were somewhat partially dedifferentiated, synthetizing both type I and II collagens. We have previously shown that it is difficult to reverse this phenotype without adding a potent chondrogenic factor such as BMP-2 [33,34,35]. However, the dedifferentiation of chondrocytes is an important phenomenon that occurs during OA, and our model therefore correctly mimics this aspect. The non-specific effect on these two matrix collagens, one typical of hyaline cartilage (type II collagen) and the other of fibrous cartilage (type I collagen), nevertheless shows that B and its combination to R could enhance or sustain matrix synthesis.

A critical point for successful cartilage regeneration is to control regeneration while preventing cartilage hypertrophy. We found that nanogels do not destabilize the chondrocyte phenotype because there is no progression towards a hypertrophic or osteoblastic phenotype. Furthermore, the two characteristic markers, type X collagen and osteocalcin, were weakly expressed. Chondrocyte hypertrophy is one of the key stages of the OA process because chondrocytes progressively become hypertrophic and enter apoptosis, with a final stage corresponding to calcification and the formation of osteophytes [56].

Finally, these functionalized nanogels were biocompatible with no cytotoxic effect and may favor metabolic activity and proliferation of eACs. In our cartilage organoid model, BR nanogels may protect cartilage against inflammation and degradation by reducing NO, MMPs, HtrA1, and IL-6 expression and have weak pro-anabolic effects on matrix markers expression. To overcome this difficulty, one approach is to update the nanogels by grafting pro-chondrogenic factors such as BMP-2 to increase the expression of type II collagen [35] and to enhance cartilage regeneration.

This study provides in vitro evidences that the functionalized nanogels we used could be of interest to improve OA treatments. Nevertheless, the in vitro culture model we used cannot recapitulate the whole articulation and body complexities of an animal model although it is a real cartilage organoid model. Hence, further in vivo studies are needed to ensure that the functionalized nanogels will be tolerated without adverse effects and to characterize the therapeutic potential of the functionalized nanogels in a clinical side.

Therefore, the CHI- and HA-based functionalized nanogels may be useful for the treatment of OA and await in vivo testing on a cohort of horses spontaneously affected by OA.

## 4. Materials and Methods

### 4.1. Nanogel Formulations

Three different nanogel formulations were developed and tested in this study: the first formulation, called the non-functionalized nanogel (CHI-HA = NG), was a combination of chitosan (CHI) (Sigma-Aldrich, Co. Oakville, ON, Canada) polysaccharides and hyaluronic acid (HA) (LifeCore Biomedical LLC, Chaska, MN, USA). The second formulation (BQ-123-CHI = B) was composed of CHI conjugated to the peptide BQ-123 (ChinaPeptides Co., Ltd., Shanghai, China), a type A endothelin receptor antagonist. The third formulation (R-954-HA = R) was composed of HA conjugated to the peptide R-954 (kind gift of Dr. Pierre Sirois, IPS Thérapeutique, Québec, Canada), a type B1 bradykinin receptor antagonist. The combination of the two functionalized formulations (BR) was studied at equimolar concentrations. Each formulation was dissolved in sterile water, and then, successive dilutions were performed with HG-DMEM to form a range of concentrations. For toxicity, metabolic activity, and proliferation experiments, the CHI-HA formulation was tested at 0.01, 0.1, 1, 10, and 100 µg/mL CHI-HA. Other formulations were tested at 1, 5, 10, 20, 30, and 60 nM receptor antagonist.

#### 4.1.1. Peptides Grafting

The two peptides, BQ-123 and R-954, were conjugated to low-molecular-weight chitosan (BQ-123-CH) and hyaluronic acid (R-954-HA), respectively, using the well-established EDC/NHS (Sigma-Aldrich Canada Co., Oakville, ON, Canada) coupling chemistry. Concentration and grafting rates were determined using spectrofluorometry (Hitachi F-2710 spectrophotometer, Hitachi High Technologies America, Inc., Pleeasanton, CA, USA) at excitation/emission wavelengths of 276/348 nm for BQ-123 and 284/335 nm for R-954. 

#### 4.1.2. Nanogel Synthesis, Purification, and Lyophilization

Nanogels (NGs) were obtained using an ionic gelation process based on a previously reported protocol [47]. Briefly, a chitosan solution (CHI, 2.5 mg/mL) in acetic acid (0.5% (*v*/*v*)) and a second solution of sodium tripolyphosphate (TPP, 1.2 mg/mL) (Alfa Aesar, Ward Hill, MA, USA) and 60 kDa sodium hyaluronate (HA, 0.8 mg/mL) were magnetically stirred overnight until complete dissolution. R-954 NG and BQ-123 NG were synthesized separately using the corresponding grafted biopolymer (either BQ-123-CHI (formulation B) for BQ-123 NG or R-954-HA (formulation R) for R-954 NG) with the same concentration of polymers as mentioned above, in aseptic conditions. The anionic TPP/HA solution was added dropwise at a constant flow rate of 4.5 mL/min to the cationic CH solution (1:2 volume ratio) under concomitant ultrasonic (US) mixing (550 Sonic Dismembrator, power 3/12, Fisher Scientific, Thermo Fisher Scientific, Waltham, MA, USA) and moderate magnetic stirring. After addition of the TPP/HA solution, US was maintained for an additional 60 s and then magnetic stirring for another 10 min. The colloidal solution changed from colorless to turbid (characteristic Tyndall effect). Nanogel suspensions were dialyzed (Spectrum, Spectra/Por^®^ 6.0, *M*_W_CO 25 kDa, Spectrum^TM^ Chemical Manufacturing Corp., New Brunswick, NJ, USA) three times at room temperature against ×100 volumes of MilliQ ultrapure water for 6 h to remove excess solubilizing agents, TPP, and unreacted polymer chains with low *M*_W_. Purified nanosuspensions were then lyophilized (ModulyoD FreezeDryer, Thermo Electro Corporation, Milford, MA, USA) with sucrose (8% *w*/*v*) (Sigma-Aldrich Canada Co., Oakville, ON, Canada) as a cryoprotective agent for a minimum of 24 h. Finally, pH as well as hydrodynamic diameters and zeta potentials were measured prior and after dialysis and lyophilization. 

#### 4.1.3. Determination of Drug Loading (DL%)

Peptide-loaded nanogels were centrifuged at 16,000 rpm, 4 °C, for 90 min to remove nanogels from the aqueous suspension medium. Supernatants were collected, and the amount of free, non-encapsulated peptide was therefore quantified by spectrofluorometry as mentioned above, the NGs masses were conversely accessed by weighting freeze-dried NGs. The drug loadings (DL%) of nanogels were then calculated as follows in Equation (1):

(1) $$\;\mathrm{DL}\;\%=\frac{W_{\mathrm{initial}}\;-\;W_{\mathrm{supernatant}}}{W_{\mathrm{NG}}+(W_{\mathrm{initial}}\;-\;W_{\mathrm{supernatant}})}\times 100$$

where W_initial_ was the initial weight of peptide introduced in the reaction mixture, W_supernatant_ was the amount of free, non-encapsuled peptide retrieved in the supernatant after centrifugation, and W_NG_ the weight of freeze-dried nanogels.

### 4.2. Isolation and Cell Culture

All cartilage samples were collected at the “Centre d’Imagerie et de Recherche sur les Affections Locomotrices Equines” (CIRALE, Goustranville, France). All procedures described in this study were approved by the Ethics Committee for Animal Experimentation (ComEth ANSES/ENVA/UPEC, 94 701 Maisons-Alfort, France; no. 15-023 (10 March 2015), no. 10-0051 (10 September 2014)). All cells used were tested and free from any bacteriological or virological contamination.

### 4.3. Culture of Equine Chondrocytes 

Equine articular chondrocytes (eACs) were isolated from biopsies of healthy equine anterior carpus on young horses (4–6 years) as previously described [37]. eACs were cultured in high-glucose DMEM (HG-DMEM, 4.5 g/L BioWest, Nuaillé, France) supplemented with 10% of fetal bovine serum (FBS), 100 IU/mL of penicillin, 100 μg/mL of erythromycin, and 0.25 mg/mL of amphotericin B (Eurobio Scientific, Courtaboeuf, France) at 37 °C in a humidified atmosphere containing 5% CO_2_. During expansion, the medium was changed twice a week. For cell passages, at 80% confluency, chondrocytes were harvested by trypsinization with trypsin–ethylenediaminetetraacetic acid (EDTA) (Eurobio Scientific, Courtaboeuf, France), counted with trypan blue to evaluate viability, and seeded at 2 × 10^4^ cells/cm^2^. 

### 4.4. Three-Dimensional Culture

As previously described [35], chondrocytes at the third passage (P3) were seeded into collagen sponges at 8 × 10^5^ cells/cm^2^ and cultured in a medium, termed as 3-dimensional (3D) medium, composed of HG-DMEM supplemented with 2% FBS and L-ascorbic acid-2-phosphate at 50 µg/mL (Sigma-Aldrich, Saint-Louis, MO, USA). The next day, scaffolds were transferred into 24-well culture plates with formulation of nanogels diluted into 3D medium with or without (IL-1β) at 10 ng/mL (Miltenyi Biotec, Bergisch Gladbach, Germany). CHI-HA formulations were tested at 0.1 and 10 µg/mL of CHI-HA and with at 5 and 30 nM of antagonists for B, R, and BR formulations. Cells were incubated in hypoxia (3% of O_2_) at 37 °C under 5% CO_2_ in a Plas-Labs basic multi-station glove box (Sigma-Aldrich, St. Louis, MO, USA) for 7 days, and the medium was changed twice a week. 

### 4.5. Evaluation of Cytotoxicity

To determine the level of cytotoxicity, cells (P3) were seeded at 2 × 10^4^ cells/cm^2^ in 96-well culture plates and incubated during 72 h in HG-DMEM supplemented of 10% FBS at 37 °C and under 5% CO_2_ atmosphere. Then, cells were treated with each nanogel formulation diluted into HG-DMEM supplemented with 5% of FBS. Experiments were carried out under normoxia (21% O_2_) and hypoxia (3% O_2_). To determine the cytotoxicity, 80 µL of culture media was transferred to a 96-well plate and incubated with assay reagent for 5 min at room temperature according to the manufacturer’s instructions (Interchim, Montluçon, France). The luminescence was read on a microplate reader (Spark Control Magellan, TECAN, Lyon, France).

### 4.6. Determination of Metabolic Activity by the XTT Test

The metabolic activity was analyzed using the XTT test directly in the plates used for the cytotoxicity experiments. This assay is based on the reduction of tetrazolium salt to orange-colored formazan only by metabolically active cells. After each time of treatment, an XTT assay was performed according to the manufacturer’s instructions (Roche, Bale, Switzerland). Optical density (OD) measurements were taken at 490 and 650 nm with a microplate reader (Spark control Magellan, TECAN).

### 4.7. Evaluation of Proliferation

To evaluate the cell proliferation, IncuCyte^®^ technology (IncuCyte S3 microscope and IncuCyte 2021A software, Sartorius, Göttingen, Germany) was used to visualize cells in real-time and quantified confluency with the IncuCyte^®^ ZOOM living cell imaging system. Cells were seeded at 2 × 10^4^ cells/cm^2^ at P3 in a 96-well culture plate and incubated during 24 h in HG-DMEM + 10% FBS at 37 °C under 21% O_2_ and 5% CO_2_ atmosphere. After 24 h, cells were treated with CHI-HA formulation (NG) (0.1 and 10 µg/mL) or BR formulations (5 and 30 nM of antagonists) with or without IL-1β (10 ng/mL, Miltenyi Biotec, Bergisch Gladbach, Germany) and diluted into HG-DMEM supplemented with 5% FBS. Then, the plate was placed into the IncuCyte^®^ apparatus, and images of cells were recorded every 2 h for a total duration of 144 h.

### 4.8. Scratch Wound Assay

eACs were seeded in monolayer at P3 (20,000 cells/cm^2^) in a 96-well-ImageLock plates (Essen BioScience, Ann Arbor, MI, USA) and were cultured for 72 h. At confluency, WoundMaker^TM^ (Essen BioScience) was used to create wounds into wells according to the manufacturer’s instructions. Treatments were then added, and wound repair was observed for 96 h using the IncuCyte^®^ ZOOM living cell imaging system (Essen BioScience). Wound surface and cell confluence were measured using ImageJ software.

### 4.9. RNA Isolation and RT-PCR

At the end of the experiments, sponges were washed twice with 0.1 M phosphate-buffered saline (Eurobio Scientific, Courtaboeuf, France), and total RNA was extracted using RNA-Solv^®^ Reagent (Ozyme, Saint Cyr l’Ecole, France) according to manufacturer’s instructions. Total RNAs (1 µg) from each sample were reverse-transcribed into cDNA using the iScript™ Reverse Transcription Supermix (Bio-Rad, Hercules, CA, USA), and real-time-PCR was performed using Go Taq^®^ Probe qPCR Master Mix on a CFX96 (Bio-Rad). The sequences of the primers used are listed in Supplementary Table S1 (Supplementary Materials), and relative gene expression was calculated using the 2^−∆∆CT^ method. Each sample was normalized versus two housekeeping genes, *ACTB* and *TUBA*. 

### 4.10. Western Blots

After treatment, sponges were rinsed twice with PBS and crushed, and total proteins were extracted at 4 °C using RIPA-lysis buffer supplemented with a protease inhibitor cocktail, as previously described [34]. To clear lysates, samples were centrifuged for 30 min (12,000× *g*) at 4 °C, and proteins were quantified using BCA protein assay according to the Bradford colorimetric procedure (Bio-Rad). Then, 10 µg of total proteins were separated in 7.5 or 10% polyacrylamide gels (Bio-Rad) and transferred to a polyvinylidene difluoride membrane (PVDF) (Bio-Rad). Unspecific binding sites of the membranes were blocked with 10% non-fat milk powder in Tris-buffered saline with 0.1% Tween (TBST) for 1 h. Then, membranes were incubated overnight at 4 °C with rabbit anti-human type I collagen (1:3000) (Novotec, Lyon, France), rabbit anti-human type II collagen (1:750) (Novotec), rabbit anti-human type IIb collagen (1:750) (Covalab, Bron, France), rabbit anti-human HtrA1 (1:3000) (AB clonal, Woburn, MA, USA), mouse anti-human type X collagen (1:1000) (Sigma-Aldrich, Saint-Louis, MO, USA), and mouse anti-human GAPDH (1:3000) (Santa Cruz Biotechnology, Dallas, TX, USA) (Supplementary Table S2 (Supplementary Materials)). The following day, membranes were washed three times and incubated with HRP-conjugated goat anti-rabbit or mouse IgG antibody (Jackson Immunoresearch, Cambridge, UK). Proteins were visualized with an enhanced chemiluminescence (Clarity Western ECL substrate, Bio-Rad) using an imager (ChemiDoc™ Touch Imaging System, Bio-Rad).

### 4.11. Nitrite Determination

To evaluate the nitric oxide (NO) production, culture supernatant from 3D cultures of eACs after 3 (D3) and 7 (D7) days of incubation was assayed using the Griess reaction (Griess Reagent Kit for Nitrite Determination, Molecular Probes, Eugene, OR, USA). Briefly, equal volumes of Griess Reagent A (sulfanilamide) and B (N-(1-naphthyl-) ethylene diamine dihydrochloride) were mixed with the culture media, and color development was measured at 548 nm using a microplate reader (Spark Control Magellan, TECAN) 30 min after incubation with Griess reagents. The amount of nitrite in the culture media was evaluated from a standard curve (0–50 μM) of sodium nitrite prepared in deionized water after subtraction of the sample blank (water).

### 4.12. Statistical Analysis

Experiments of cytotoxicity, metabolic activity, and proliferation were repeated at least three times with three different cell strains. Other experiments used cartilage samples from at least four strains of horses. Values are reported as means ± SEM. Statistical analyses were carried out after a Shapiro–Wilk test to evaluate if data followed a normal distribution, using Student’s *t*-tests or Mann–Whitney tests to determine significant differences between two groups or using two-way ANOVA to compare different parameters. Statistical analyses were done using Prism (GraphPad Prism 8, San Diego, CA, USA), and a *p*-value of ≤0.05 was considered to be significant.

## Figures and Tables

**Table 1:** BQ-123-CHI and R-954-HA nanogels physicochemical characteristics. Mean and standard deviations of hydrodynamic diameter (D_H_ (nm)), polydispersity index (PdI), and zeta potential (ZP) were measured by dynamic light scattering and electrophoretic light scattering, respectively. The amount of free, non-encapsulated peptide was quantified by spectrofluorometry and used to determine the drug loading (DL). *n* = 2 batches for BQ-123-CHI, and *n* = 3 batches for R-954-HA.

	Size (D_H_, nm)	PdI	ZP (mV)	DL (%)
**BQ-123-CHI**	733 ± 169	0.60 ± 0.3	47 ± 5	1.35–2.60
**R-954-HA**	429 ± 52	0.29 ± 0.0	51 ± 8	0.30–0.63

## Data Availability

The data presented in this study are available in Supplementary Materials.

## Supplementary Materials

The following supporting information can be downloaded at: https://www.mdpi.com/article/10.3390/ijms23168949/s1.
