# Supplementary material for: Functionalized Nanogels with Endothelin-1 and Bradykinin Receptor Antagonist Peptides Decrease Inflammatory and Cartilage Degradation Markers of Osteoarthritis in a Horse Organoid Model of Cartilage
Source: Int J Mol Sci. 2022 Aug 11;23(16):8949. doi: 10.3390/ijms23168949 (PMC9408731; doi:10.3390/ijms23168949)
Supplement: Supplementary file 1 [file ijms-23-08949-s001.zip › ijms-1816245-supplementary.pdf]

Functionalized nanogels with endothelin-1 and bradykinin receptor antagonists peptides decrease inflammatory and cartilage degradation markers of osteoarthritis in a horse organoid model of cartilage

Aurélie Cullier [1], Frédéric Cassé 1[,†], Seng Manivong [2,3,†], Romain Contentin [1,‡], Florence Legendre [1,‡], Aracéli Garcia Ac [3,4], Pierre Sirois [5], Gaëlle Roullin [3], Xavier Banquy [3], Florina Moldovan [2,*], Lélia Bertoni [6], Fabrice Audigié [6], Philippe Galéra 1 and Magali Demoor [1,*]

1. Normandie Univ, UNICAEN, BIOTARGEN, 14000 Caen, France
2. Research Center of CHU Sainte Justine and Faculty of Dentistry, Université de Montréal, Montreal, QC (H3T 1C5), Canada
3. Faculty of Pharmacy, Université de Montréal, Montréal, QC (H3T 1J4), Canada
4. TransMedtech Institute (NanoBio Technology Platform), Montréal, QC (H3T 1J4), Canada
5. Department of Microbiology and Immunology, Faculty of Medicine, Université de Laval, Québec, QC (G1V 4G2), Canada
6. Center of Imaging and Research on Locomotor Affections in Equines, Veterinary School of Alfort, 14430 Goustranville, France

†, ‡ Equal contribution

*Correspondence: florina.moldovan@umontreal.ca; magali.demoor@unicaen.fr

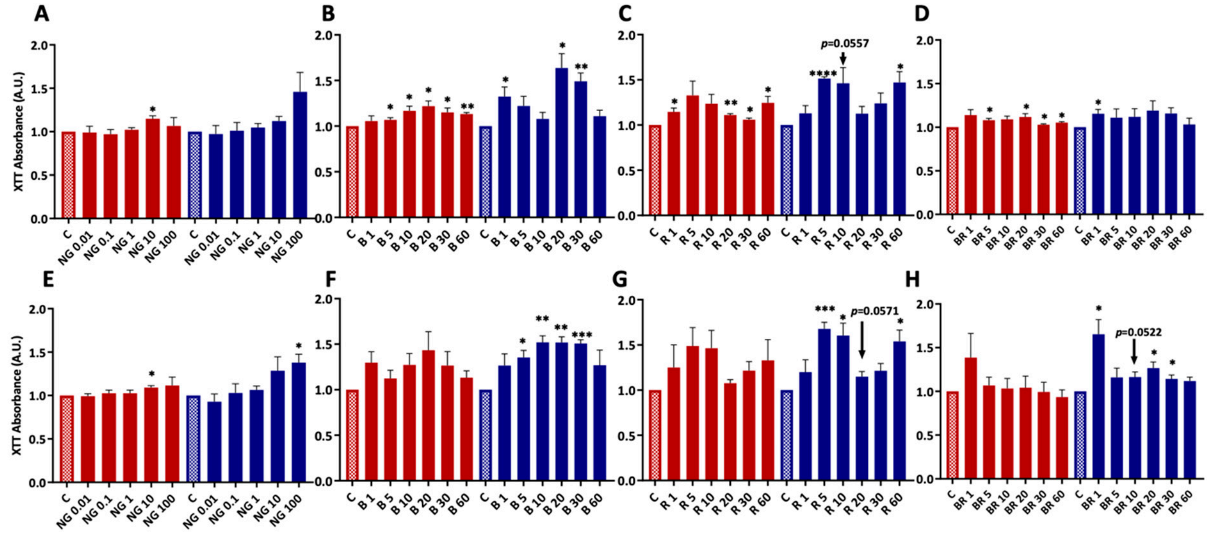

Figure S1. Effects of nanogels on the viability and proliferation of equine articular chondrocytes (eACs). eACs were amplified and seeded at the third passage in monolayer (20 000 cells/cm^2). At 80% confluence, cells were treated with nanogels formulations, NG at 0.01, 0.1, 1, 10 or 100 µg/mL, NG-BQ-123 (B), NG-R-954 (R) and BR at 1, 5, 10, 20, 30 or 60 nM) and then incubated in normoxia **(red)** or in hypoxia **(blue)** for 24h **(A, B, C, D)** and 48h **(E, F, G, H)**. Control (without nanogels) was included. The levels of formazan were measured in the culture media at the end of each incubation time (XTT kit, Roche). Data are represented as histograms (n=3). Student *t*-tests (* $p < 0.05$, ** $p < 0.01$, *** $p < 0.001$, **** $p < 0.001$) were used to compare each treatment with control. Results were normalized to the control. NG: Non-functionalized nanogel, B: BQ-123-CHI, R: R-954-HA, BR: Equimolar combination of BQ-123-CHI and R-954-HA, C: Control, A.U.: Arbitrary Units.

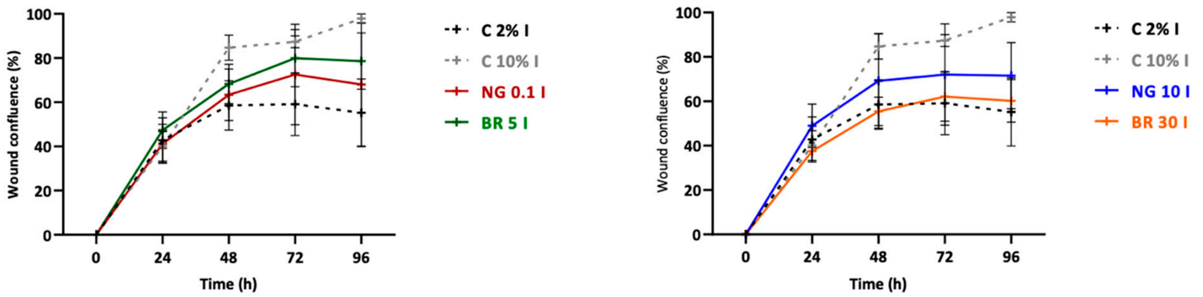

Figure S2. Effects of nanogels on wound filling in presence of IL-1β. Equine articular chondrocytes (eACs) were amplified and seeded at the third passage in monolayer (20 000 cells/cm^2). At 100% of confluence, a wound filling was assayed in each well and nanogel treatments were added to cells, 0.1 and 10 µg/mL NG, 5 and 30 nM BR (in the presence of 2% FBS (C 2%)), with IL-1β (10 ng/mL) and proliferation was followed by IncuCyte®. The cells were also incubated in the presence of 10% FBS (C 10%). At the end of the incubation period, confluence and wound area were analyzed with ImageJ software. Data are represented as curves (n=3). NG: Non-functionalized nanogel, BR: Equimolar combination of BQ-123-CHI and R-954-HA, C: Control, I: IL-1β.

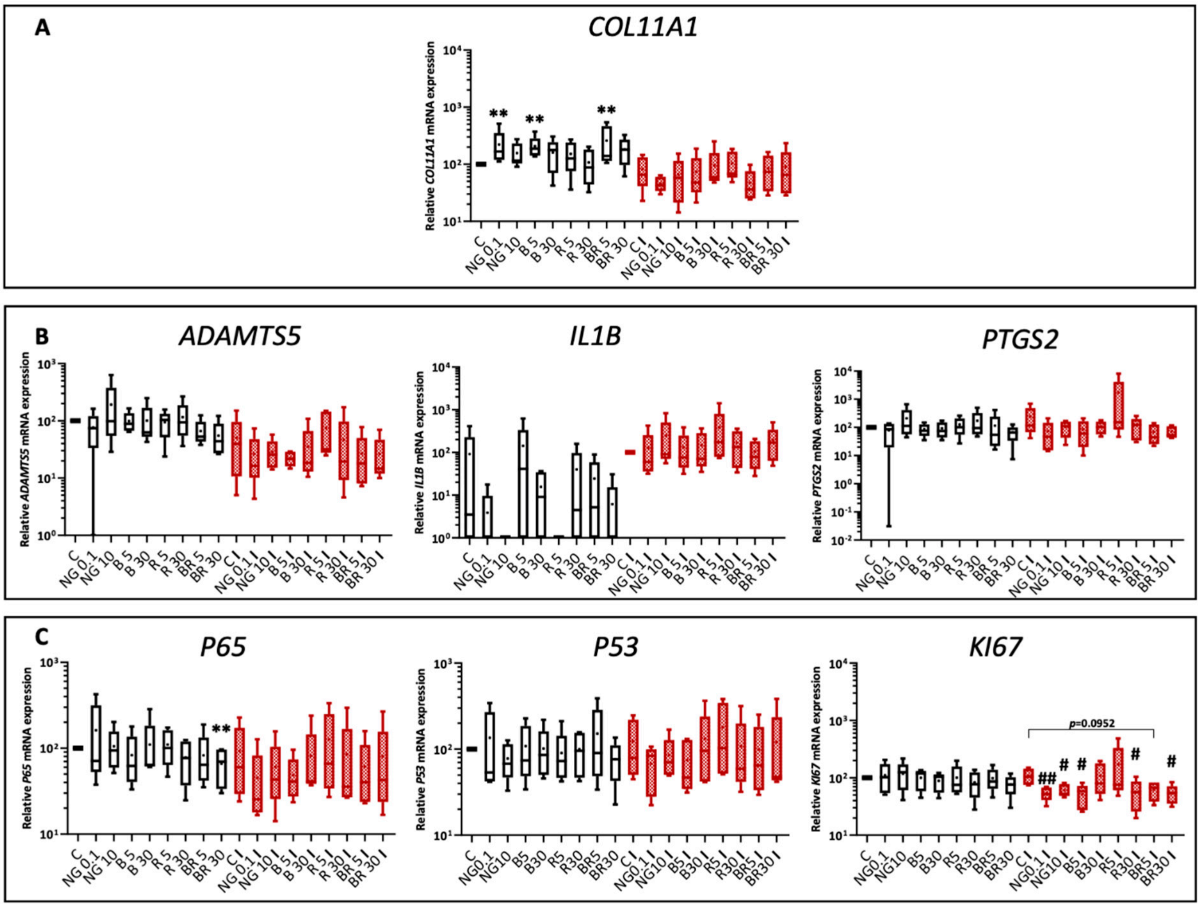

Figure S3. Other markers gene expression analysis of equine articular chondrocytes (eACs) cultured with nanogels and IL-1β. eACs were grown in type I/III collagen sponges and then incubated for 7 days in hypoxia in the absence (C) or presence of nanogel formulations (NG at 0.1 and 10 µg/mL, B, R and BR at 5 and 30 nM) and in the absence **(black)** or presence **(red)** of IL-1β (C I) (10 ng/mL). Transcript expression of some matrix **(A)**, catabolic and inflammatory **(B)** and others **(C)** markers are shown in arbitrary units. The results are shown as box plots (n=5). Statistical analyses were performed by using a Mann-Whitney test, significantly (# $p < 0.05$, **, ## $p < 0.01$) different from C (*) and C I (#). NG: Non-functionalized nanogel, B: BQ-123-CHI, R: R-954-HA, BR: Equimolar combination of BQ-123-CHI and R-954-HA, C: Control, I: IL-1β.

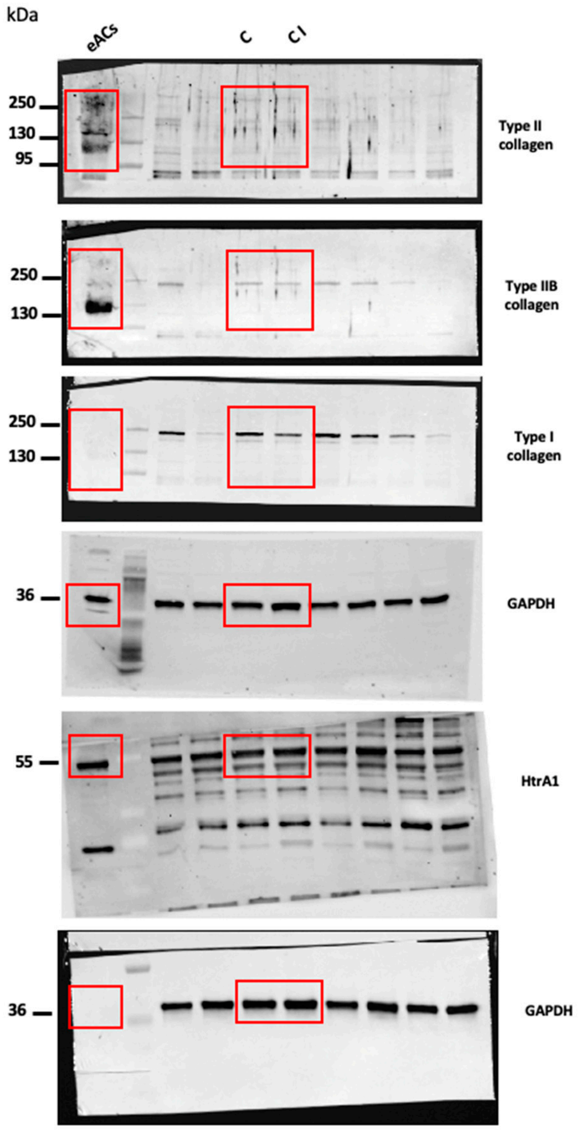

Figure S4. Complete gel and PVDF membranes analyzed in the western blots. For the western blots presented in Figure 9A, the entire images of the membranes captured with the Chemidoc MP Imaging System (Bio-Rad) are shown. The molecular weight marker (MW, kDa) is indicated on the left side of the images. Red boxes indicate the cropped images shown in Figure 9A.

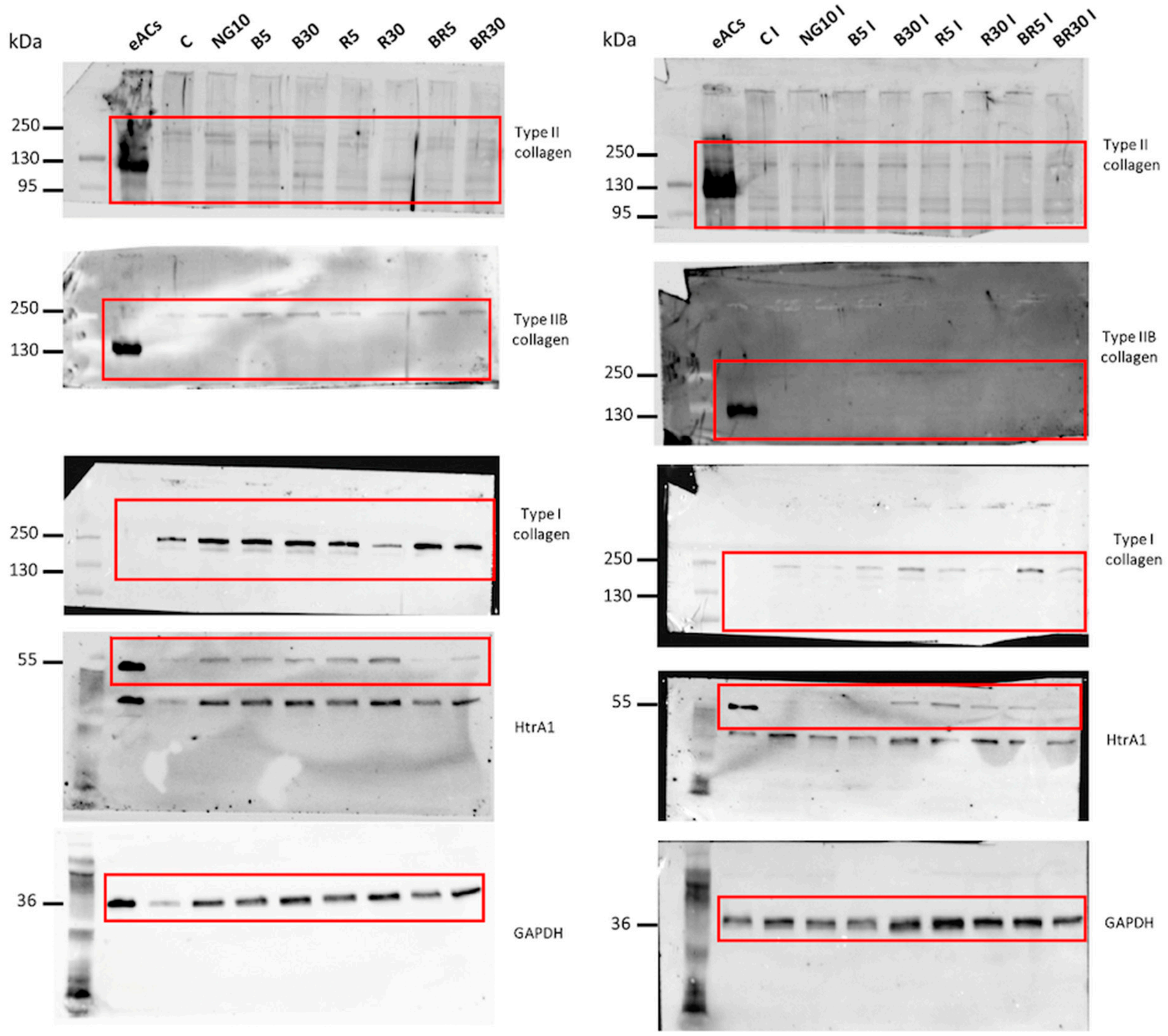

Figure S5. Complete gel and PVDF membranes analyzed in the western blots. For the western blots presented in Figure 9B and C, the entire images of the membranes captured with Chemidoc MP Imaging System (Bio-Rad) are shown. The molecular weight marker (MW, kDa) is indicated on the left side of the images shown. Red boxes indicate the cropped images shown in Figure 9B and C.

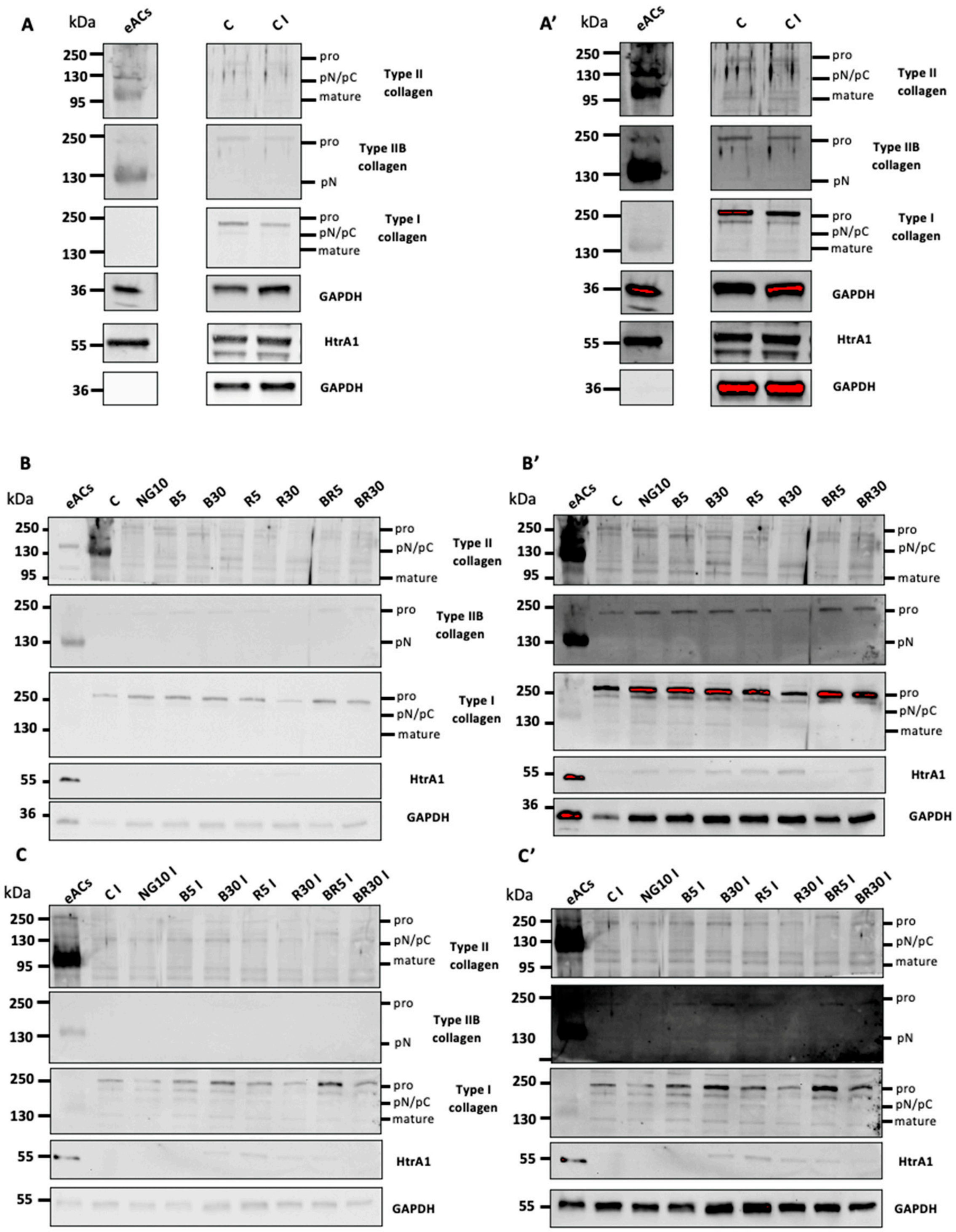

Figure S6. Under- and overexposure of the blots shown in Figure 9. Under-exposure (A, B and C) and over-exposure (A', B' and C') of blots shown in Figure 9A, B and C. The molecular weight marker (kDa) is indicated on the left side of the images.

Supplementary Table S1. Sequences of the primers used in RT-qPCR

Gene	Forward sequence	Reverse sequence
ACAN	TGT CAA CAA CAA TGC CCA AGA C	CTT CTT CCG CCC AAA GGT CC
ACTB	GAT GAT GAT ATC GCC GCG CTC	TGC CCC ACG TAT GAG TCC TT
ADAMTS5	AAG GGA CAC CAT GTG GCA AA	CCC ACA TGA GCG AGA ACA CT
ALPL	GAC ATG ACC TCC CAG GAA GA	GCA GTG AAG GGC TTC TTG TC
BDKRB1	GGA GAC CAA TGT TCC TGT CTG T	TGG CAT TTG GAG GCA AGA GC
BGLAP	TCC TTT GGG GTT TGG CCT AC	GCC TGT GAG ACA AAG GAG GG
COL1A1	TGC CGT GAC CTC AAG ATG TG	CGT CTC CAT GTT GCA GAA GA
COL1A2	CCA GAG TGG AGC AGC GGT TA	GGG ATG TTT TCA GGT TGA GCC
COL2A1	GGC AAT AGC AGG TTC ACG TAC A	CGA TAA CAG TCT TGC CCC ACT T
COL10A1	GCA CCC CAG TAA TGT ACA CCT ATG	GAG CCA CAC CTG GTC ATT TTC
COL11A1	TTG CTG ATG GGA AGT GGC AT	GCT GCT TTG GGG TCA CCT AT
EDNRA	ATT TAA GCT GCT AGC TGG GC	ATC CCG ATT CCC TGA ACA CG
PTGS2	CGA GGT CCA GCT TTC ACC A	GCG GAT ACA CCT CGC CAT T
PRG4	CTA CCA CCC AAC GCA ACA AA	ACT GTT GTC TCC TTA TTG GGT
HTRA1	GGA CTT CAT GTT TCC CTC AA	GTT CTG CTG AAC AAG CAA CA
IL1B	CCC ACA CCA GTG ACA TGA TGA	TCC TCC TCA AAG AAC AGG TCA TTC
IL6	GGA TGC TTC CAATCT GGG TTC AAT	TCC GAA AGA CCA GTG GTG ATT TT
IL8	GCC ACA CTG CGA AAA CTC A	GCA CAA TAA TCT GCA CCC ACT TT
IL18	GAA ATC AAC CTG TGT TTG AGG ATA TG	TCA CAG AGA TGG TTA CCG CTA GAC
INOS	TTT GGC TGG TCC CCC GAT TT	GCC AGC GTT TCC GAT TTT CC
KI67	AAG CTG CAC GTT CAT GGA GA	ACC CAC AGT TCT TCC TCC GA
MMP1	CGA AGG GAA CCC TCG GTG GGA	TGG CCT GGT CCA CAT CTG CTC
MMP3	GAG GAA ATG AGG AAC AAG CGG	GAG GGA AAC CCA GAG TGT GGA
MMP13	TGA AGA CCC GAA CCC TAA ACA T	GAA GAC TGG TGA TGG CAT CAA G
P53	CAC CTG AGG TTG GCT CTG AC	GCA CAA ACA CGC ACC TCA AA
P65	CAC GGA TAC CAC CAA GAC CC	GTC TGG ATG CGC TGA CTG AT
SOX9	CAA GAA GGA CCA CCC GGA CTA	GGA GAT GTG TGT CTG CTC CGT
SPP1	GAG ACA CGT ATG ATG GCC GA	GCT GTC CCA ATC AGA AGC CA
TUBA	GAG TTT TCT GAG GCC CGT GA	GTC TCC CTG TAA AAG CAG CAC

Supplementary Table S2. Antibodies used in the western blots

Antibody	Supplier	Dilution
**Type IIB collagen**	Covalab, Villeurbanne, France	1:750
**Type II collagen**	Novotec, Bron, France	1:750
**Type I collagen**	Novotec, Bron, France	1:3000
**HtrA1**	ABGENT, San Diego CA, USA	1:3000
**GAPDH**	Santa Cruz Biotechnology, Dallas TX, USA	1:3000
